# Supplementary material for: End Sequence Analysis Toolkit (ESAT) expands the extractable information from single-cell RNA-seq data
Source: Genome Res. 2016 Oct;26(10):1397–410. doi: 10.1101/gr.207902.116 (PMC5052061; doi:10.1101/gr.207902.116)
Supplement: Supplemental Material [file supp_gr.207902.116_Supp_Materials.tar.gz › ESAT-esat_v0.1/doc/html/classbroad_1_1pda_1_1seq_1_1fastq_1_1_fastq_sequence.html]

ESAT: broad.pda.seq.fastq.FastqSequence Class Reference


ESAT


- Main Page
- Related Pages
- Packages
- Classes
- Files

- Class List
- Class Index
- Class Hierarchy
- Class Members

All Classes Namespaces Files Functions Variables Enumerator Pages

- broad
- pda
- seq
- fastq
- FastqSequence

Public Member Functions |
List of all members

broad.pda.seq.fastq.FastqSequence Class Reference

|  |  |
| --- | --- |
| Public Member Functions | |
|  | FastqSequence (String name, String sequence, String description, String quality) |
|  | |
| String | getName () |
|  | |
| String | getSequence () |
|  | |
| String | getDescription () |
|  | |
| void | setName (String name) |
|  | |
| void | setDescription (String descr) |
|  | |
| String | toString () |
|  | |
| void | write (BufferedWriter bw) throws IOException |
|  | |
| void | removeAtSymbolFromName () |
|  | |
| FastqSequence | trimEnds (char letter) |
|  | |
| FastqSequence | trimEndBases (int numOfBasesToTrim) |
|  | |
| FastqSequence | trimStartBases (int numOfBasesToTrim) |
|  | |
| FastqSequence | trimBeginning (char letter) |
|  | |
| FastqSequence | trimFirstNBPs (int n) |
|  | |
| String | getFirstNBPs (int n) |
|  | |
| int | getLength () |
|  | |
| String | toFasta () |
|  | |
| String | toFasta (int readNum) |
|  | |
| String | toFastq () |
|  | |
| boolean | isPolyA () |
|  | |
| boolean | isPartialPolyA (int polyN) |
|  | |
| FastqSequence | trimPolyA () |
|  | |
| FastqSequence | trimLinker (String linkerSeq) |
|  | |

## Constructor & Destructor Documentation

|  |  |  |  |
| --- | --- | --- | --- |
| broad.pda.seq.fastq.FastqSequence.FastqSequence | ( | String | *name*, |
|  |  | String | *sequence*, |
|  |  | String | *description*, |
|  |  | String | *quality* |
|  | ) |  |  |

Here is the caller graph for this function:

## Member Function Documentation

|  |  |  |  |  |
| --- | --- | --- | --- | --- |
| String broad.pda.seq.fastq.FastqSequence.getDescription | ( |  | ) |  |

Here is the caller graph for this function:

|  |  |  |  |  |  |
| --- | --- | --- | --- | --- | --- |
| String broad.pda.seq.fastq.FastqSequence.getFirstNBPs | ( | int | *n* | ) |  |

|  |  |  |  |  |
| --- | --- | --- | --- | --- |
| int broad.pda.seq.fastq.FastqSequence.getLength | ( |  | ) |  |

Here is the caller graph for this function:

|  |  |  |  |  |
| --- | --- | --- | --- | --- |
| String broad.pda.seq.fastq.FastqSequence.getName | ( |  | ) |  |

|  |  |  |  |  |
| --- | --- | --- | --- | --- |
| String broad.pda.seq.fastq.FastqSequence.getSequence | ( |  | ) |  |

|  |  |  |  |  |  |
| --- | --- | --- | --- | --- | --- |
| boolean broad.pda.seq.fastq.FastqSequence.isPartialPolyA | ( | int | *polyN* | ) |  |

Here is the call graph for this function:

|  |  |  |  |  |
| --- | --- | --- | --- | --- |
| boolean broad.pda.seq.fastq.FastqSequence.isPolyA | ( |  | ) |  |

Here is the call graph for this function:

Here is the caller graph for this function:

|  |  |  |  |  |
| --- | --- | --- | --- | --- |
| void broad.pda.seq.fastq.FastqSequence.removeAtSymbolFromName | ( |  | ) |  |

|  |  |  |  |  |  |
| --- | --- | --- | --- | --- | --- |
| void broad.pda.seq.fastq.FastqSequence.setDescription | ( | String | *descr* | ) |  |

|  |  |  |  |  |  |
| --- | --- | --- | --- | --- | --- |
| void broad.pda.seq.fastq.FastqSequence.setName | ( | String | *name* | ) |  |

|  |  |  |  |  |
| --- | --- | --- | --- | --- |
| String broad.pda.seq.fastq.FastqSequence.toFasta | ( |  | ) |  |

|  |  |  |  |  |  |
| --- | --- | --- | --- | --- | --- |
| String broad.pda.seq.fastq.FastqSequence.toFasta | ( | int | *readNum* | ) |  |

|  |  |  |  |  |
| --- | --- | --- | --- | --- |
| String broad.pda.seq.fastq.FastqSequence.toFastq | ( |  | ) |  |

|  |  |  |  |  |
| --- | --- | --- | --- | --- |
| String broad.pda.seq.fastq.FastqSequence.toString | ( |  | ) |  |

|  |  |  |  |  |  |
| --- | --- | --- | --- | --- | --- |
| FastqSequence broad.pda.seq.fastq.FastqSequence.trimBeginning | ( | char | *letter* | ) |  |

Here is the call graph for this function:

|  |  |  |  |  |  |
| --- | --- | --- | --- | --- | --- |
| FastqSequence broad.pda.seq.fastq.FastqSequence.trimEndBases | ( | int | *numOfBasesToTrim* | ) |  |

Trims the last numBasesToTrim bases of the fastq record and returns the nucleotides trimmed.

Parameters
:   |  |  |
    | --- | --- |
    | numOfBasesToTrim |  |

Returns

Here is the call graph for this function:

|  |  |  |  |  |  |
| --- | --- | --- | --- | --- | --- |
| FastqSequence broad.pda.seq.fastq.FastqSequence.trimEnds | ( | char | *letter* | ) |  |

Here is the call graph for this function:

|  |  |  |  |  |  |
| --- | --- | --- | --- | --- | --- |
| FastqSequence broad.pda.seq.fastq.FastqSequence.trimFirstNBPs | ( | int | *n* | ) |  |

Here is the call graph for this function:

|  |  |  |  |  |  |
| --- | --- | --- | --- | --- | --- |
| FastqSequence broad.pda.seq.fastq.FastqSequence.trimLinker | ( | String | *linkerSeq* | ) |  |

Here is the call graph for this function:

|  |  |  |  |  |
| --- | --- | --- | --- | --- |
| FastqSequence broad.pda.seq.fastq.FastqSequence.trimPolyA | ( |  | ) |  |

Here is the call graph for this function:

|  |  |  |  |  |  |
| --- | --- | --- | --- | --- | --- |
| FastqSequence broad.pda.seq.fastq.FastqSequence.trimStartBases | ( | int | *numOfBasesToTrim* | ) |  |

Trims the first numBasesToTrim bases of the fastq record and returns the nucleotides trimmed.

Parameters
:   |  |  |
    | --- | --- |
    | numOfBasesToTrim |  |

Returns

Here is the call graph for this function:

|  |  |  |  |  |  |
| --- | --- | --- | --- | --- | --- |
| void broad.pda.seq.fastq.FastqSequence.write | ( | BufferedWriter | *bw* | ) | throws IOException |

---

The documentation for this class was generated from the following file:

- java/broad/pda/seq/fastq/FastqSequence.java


---

Generated on Fri Aug 15 2014 15:11:02 for ESAT by  

 1.8.7
